# Impact of Malaria Infection on the Diagnostic Performance of Adipsin for Preeclampsia in Pregnancy: A Case‐Control Study

**DOI:** 10.1155/jp/4688006

**Published:** 2026-01-13

**Authors:** Bismark Opoku Mensah, Ernestina Obenewaa Anim, Linda Ahenkorah Fondjo, Akwasi Owusu Manu

**Affiliations:** ^1^ Department of Biological Sciences, University of Worcester, Worcester, UK, worcester.ac.uk; ^2^ Department of Nursing, All Nations University, Koforidua, Ghana; ^3^ Department of Molecular Medicine, KNUST School of Medicine and Dentistry, Kumasi, Ghana; ^4^ Department of Medical Diagnostics, College of Health and Well-Being, Kintampo, Ghana

**Keywords:** adipsin, biomarkers, complement factor D, complement system, inflammatory cytokines, malaria in pregnancy, *Plasmodium falciparum*, preeclampsia

## Abstract

**Background:**

Malaria and preeclampsia are major pregnancy‐related complications that share overlapping complement and inflammation‐mediated pathways. Although adipsin has been proposed as a diagnostic biomarker for preeclampsia, its diagnostic performance in the context of concurrent malaria infection remains poorly understood. This study investigated the impact of malaria infection on plasma adipsin levels and evaluated its diagnostic performance for preeclampsia.

**Methods:**

This case‐control study included 200 pregnant women between 20 and 42 weeks of gestation, stratified into four groups: normotensive without malaria, normotensive with malaria, preeclamptic with malaria, and preeclamptic without malaria (*n* = 50 per group). Plasma adipsin, C3a, C5a, TNF‐*α*, IL‐6, IL‐8 and IFN‐*γ* were measured using commercial ELISA kits. Malaria infection was confirmed with Giemsa‐stained blood smears. Data were analysed using Statistical Package for the Social Sciences (SPSS) Version 27.0.

**Results:**

Amongst the participants enrolled, malaria infection was present in 50% and preeclampsia in 50% of the sample. Plasma adipsin levels were significantly elevated in malaria‐infected and preeclamptic participants (*p* < 0.001), with the highest concentrations observed in participants with coexisting preeclampsia and malaria infection. Plasma adipsin showed strong positive correlations with C5a (*ρ* = 0.695), IL‐6 (*ρ* = 0.687), and TNF‐*α* (*ρ* = 0.645), and moderate correlations with malaria parasite density (*ρ* = 0.553), IL‐8 (*ρ* = 0.475) and C3a (*ρ* = 0.437) (*p* < 0.001 for all). Multivariable regression showed that preeclampsia and malaria independently elevated plasma adipsin levels, with a significant negative interaction between the two conditions (*p* < 0.001). ROC analysis showed reduced diagnostic specificity for preeclampsia in malaria‐infected participants (62.1%, AUC = 0.719, *p* = 0.02) compared with malaria‐negative participants (87.9%, AUC = 0.823, *p* < 0.001).

**Conclusion:**

*Plasmodium falciparum* infection significantly alters plasma adipsin levels, reducing its diagnostic specificity for preeclampsia. Malaria‐adjusted reference thresholds may be necessary when considering adipsin as a biomarker in endemic regions.

## 1. Background

Malaria and preeclampsia are two major pregnancy‐related complications that share similar immunological and pathological features [1–3]. Malaria, caused by *Plasmodium falciparum*, is a leading cause of maternal morbidity and mortality, contributing to adverse maternal and foetal outcomes [4]. Preeclampsia, a hypertensive disorder of pregnancy, similarly threatens maternal and foetal health and is characterized by endothelial dysfunction, hypertension, and placental insufficiency [5]. Both conditions involve abnormal immune activation, complement system dysregulation, and placental inflammation [6, 7].

Adipsin, also known as complement factor D, is a serine protease in the alternative complement pathway. It plays a significant role in complement activation, in both malaria infection and preeclampsia [8, 9]. As a key component in the formation of C3 convertase, adipsin amplifies immune responses in malaria infection and contributes to endothelial damage, immune dysregulation, and impaired placental development in preeclampsia [9, 10].

Recent studies have highlighted adipsin as a potential biomarker for preeclampsia due to its involvement in endothelial dysfunction and complement activation [11, 12]. Elevated adipsin levels have been reported in women with preeclampsia, suggesting its potential as a diagnostic biomarker for the condition [11, 13, 14]. Similarly, elevated adipsin levels have been observed in pregnant women infected with *P. falciparum* [15]. This suggests that malaria infection could significantly increase the levels of this biomarker in women who have preeclampsia coexisting with malaria infection. Inflammatory cytokines and complement fractions involved in preeclampsia are also observed in malaria infection [16]. However, the impact of malaria‐induced immune activation on adipsin secretion and its diagnostic performance in differentiating preeclampsia from malaria‐induced endothelial dysfunction remains poorly understood.

Despite growing evidence supporting adipsin as a biomarker for preeclampsia, its utility in malaria‐endemic settings is uncertain due to the overlap in immunological and inflammatory pathways between the two conditions. Given the shared pathophysiological mechanisms between malaria and preeclampsia, and the emerging interest in adipsin as a diagnostic tool for preeclampsia, there is a need to investigate how malaria infection influences adipsin levels in pregnant women and assess its potential as a biomarker in malaria‐endemic regions. Furthermore, it is important to evaluate the performance of adipsin in the context of malaria infection during pregnancy, particularly in regions where malaria is endemic.

This study thus examined the relationship between malaria infection, adipsin levels, and preeclampsia, comparing adipsin levels in preeclamptic women with and without malaria.

## 2. Materials and Methods

### 2.1. Study Area

This study was conducted at the Eastern Regional Hospital and Providence Medical Centre, both located in the Eastern Region of Ghana. The region is characterised by perennial malaria transmission with seasonal peaks and a high burden of malaria in pregnancy. Eastern Regional Hospital serves as a major referral centre providing antenatal, obstetric, and laboratory diagnostic services to a mixed urban and peri‐urban population, whereas Providence Medical Centre provides primary and specialist maternal healthcare.

### 2.2. Study Design and Population

This hospital‐based, case‐control study was conducted at the Eastern Regional Hospital and Providence Medical Centre, Ghana, from January 2024 to March 2025. The study population consisted of pregnant women aged 18 years or older with gestational ages ranging from 20 to 42 weeks, who attended antenatal care or were in‐patients at the healthcare facilities at the time of the study. Participants were recruited consecutively and categorised into four distinct groups: normotensive without malaria (Group 1), normotensive with malaria (Group 2), preeclamptic with malaria (Group 3), and preeclamptic without malaria (Group 4). The classification of the preeclamptic group was based on clinical assessment and the criteria established by the International Society for the Study of Hypertension in Pregnancy (ISSHP). Women in the malaria‐infected groups were confirmed by clinical examination and health records to have no other comorbidities. Similarly, participants in the preeclamptic groups were confirmed to have no other comorbid conditions.

### 2.3. Ethical Considerations

The study protocol was approved by the Committee on Human Research, Publications, and Ethics at Kwame Nkrumah University of Science and Technology (Approval ID: CHRPE/AP/1206/24). Prior to enrolment, all participants were provided with detailed study information, and written informed consent was obtained in accordance with ethical guidelines.

### 2.4. Sample Size and Eligibility Criteria

A total of 200 pregnant women were enrolled in the study using a consecutive sampling technique. Participants were equally allocated into four groups (*n* = 50 per group). The sample size was determined using G ^∗^Power Version 3.1.9.7 [17], based on an estimated moderate effect size (*f* = 0.25), a significance level (*α*) of 0.05, and statistical power of 80%, which resulted in a minimum required sample size of 180 participants for between‐group comparisons.

Eligible participants were pregnant women aged ≥ 18 years with singleton pregnancies between 20 and 42 weeks of gestation. Group classification was based on clinical assessment and standardised diagnostic criteria. Preeclampsia was diagnosed according to the ISSHP guidelines: systolic blood pressure (SBP) ≥ 140 mmHg and/or diastolic blood pressure ≥ 90 mmHg, measured on two occasions at least 4 h apart, with accompanying proteinuria defined as a protein‐to‐creatinine ratio ≥ 0.3 mg/mg.

Women in the preeclamptic groups (with or without malaria) were excluded if they had HIV, gestational diabetes, renal disease, or any other comorbidity. Only participants infected with *P. falciparum* and free from other comorbidities were included in the malaria groups. The normotensive with malaria group consisted of women with confirmed malaria but without evidence of hypertension, proteinuria or any other comorbid conditions. The preeclamptic with malaria group included women who met the criteria for preeclampsia and had confirmed malaria infection. The normotensive nonmalaria controls were required to be free from both malaria and preeclampsia and to have no chronic illness, concurrent infection, multiple gestation or other pregnancy complication. Across all four groups, participants receiving antimalarial, antihypertensive or anti‐inflammatory medications were excluded.

### 2.5. Clinical Assessment and Data Collection

Sociodemographic and obstetric data, including maternal age, gravidity, parity, primigravida status, gestational age (confirmed by early ultrasound and last menstrual period) and antenatal history were collected using structured questionnaires. Blood pressure was measured by trained midwives using a calibrated automated sphygmomanometer (Omron HEM‐907XL, Omron Healthcare, Kyoto, Japan). Two readings were taken at least 4 h apart after 5 min of rest in a seated position, and the average was used for diagnostic classification. Body weight and height were measured using a digital scale and stadiometer (SECA 877 and SECA 213, SECA GmbH, Hamburg, Germany).

### 2.6. Sample Collection and Processing

Venous blood (10 mL) was collected from each participant into K2‐EDTA anticoagulant tubes (BD Vacutainer, Becton Dickinson, United States). All samples were collected in the morning between 9:00 and 11: am. For normotensive participants, samples were collected during routine antenatal visits, whereas for the preeclamptic group, samples were taken at the time of diagnosis. All participants were between 20 and 42 weeks of gestation at the time of sample collection. Samples were placed on ice and processed within 1 h after collection. Plasma was separated by centrifugation at 1500 × g for 15 min at 4°C. The resulting plasma was then dispensed into cryovials and stored at –80°C until further analysis.

### 2.7. Laboratory Analysis

#### 2.7.1. Quantification of Plasma Adipsin, Complement Activation Fragments and Inflammatory Cytokines

Plasma levels of adipsin were measured with the Human Adipsin ELISA Kit (RayBiotech, United States). C3a and C5a were quantified with the human complement C3a and C5a ELISA Kits (Invitrogen, Thermo Fisher Scientific, United States). TNF‐*α*, IL‐6, IL‐8 and IFN‐*γ* were measured using the human TNF‐alpha ELISA Kit, human IL‐6 ELISA Kit, Human IL‐8 ELISA Kit, and human IFN‐gamma ELISA Kit, respectively (Invitrogen, Thermo Fisher Scientific, United States). All assays were performed following manufacturers′ protocols. Plasma samples were thawed only once prior to analysis, and absorbances were read at 450 nm using a SpectraMax iD3 microplate reader (Molecular Devices, San Jose, California, United States).

#### 2.7.2. Malaria Diagnosis and Parasitaemia Quantification

All participants were screened for malaria infection using the CareStart Malaria HRP2/pLDH Combo rapid diagnostic test (Access Bio Inc., United States), followed by microscopy of Giemsa‐stained thick and thin blood films. Parasitaemia was quantified by counting *P. falciparum* parasites against 200 leukocytes, and parasite density was calculated based on an assumed white blood cell (WBC) count of 8000/*μ*L. Microscopic examinations were performed independently by two experienced microscopists, with discordant results resolved by a third reviewer.

### 2.8. Statistical Analysis

All statistical analyses were conducted using Statistical Package for the Social Sciences (SPSS) Version 27.0 (IBM Corp., Armonk, New York, United States). Categorical variables were reported as frequencies and percentages. Normality of continuous variables was assessed using the Shapiro–Wilk test. Normally distributed variables were presented as mean ± standard deviation (SD), whereas non‐normally distributed variables were summarised as median and interquartile range (IQR). Group comparisons were conducted using analysis of variance (ANOVA) for normally distributed variables and the Kruskal–Wallis test for non‐normally distributed variables. Associations between adipsin and immunological markers (C3a, C5a, TNF‐*α*, IL‐6, IL‐8 and IFN‐*γ*), as well as malaria parasite density, were evaluated using Spearman′s rank correlation coefficient (*ρ*).

Covariates for multivariable regression models were selected a priori based on biological plausibility and previously reported associations with complement activation and preeclampsia. Maternal age, body mass index (BMI), gestational age and malaria parasite density were included in all adjusted models. Although several biomarker variables were skewed, regression diagnostics confirmed that model assumptions were satisfied without log‐transformation; therefore, untransformed biomarker values were retained.

Diagnostic performance of plasma adipsin for preeclampsia was assessed using receiver operating characteristic (ROC) curve analysis, with area under the curve (AUC), sensitivity, specificity and optimal threshold values reported. A *p* value < 0.05 was considered statistically significant.

## 3. Results

The study recruited 200 participants from the 246 women approached, yielding a response rate of 81.3%.

### 3.1. Sociodemographic Characteristics

Participants had a mean maternal age of 30.2 ± 6.0 years and a mean gestational age of 24.8 ± 6.3 weeks. The average BMI was 27.5 ± 4.5 kg/m^2^, and mean systolic and diastolic blood pressures were 131.0 ± 22.9 mmHg and 84.5 ± 17.0 mmHg, respectively. Gravidity and parity had median values of 3 (IQR: 2–3) and 2 (IQR: 1–3), respectively.

The maternal age, gravidity, parity, BMI and primigravida status did not show statistical difference across the groups (*p* > 0.05). However, gestational age showed a statistically significant difference across the groups (*p* = 0.030). The control group had the lowest gestational age (22.96 ± 5.29 weeks), whereas the preeclamptic group with malaria infection had the highest gestational age (25.87 ± 7.40 weeks). Post hoc analysis revealed that preeclamptic women with malaria had a significantly higher gestational age compared with normotensive women without malaria (*p* < 0.05).

There was a statistically significant difference in both SBP and diastolic blood pressure (DBP) across groups (*p <0.001* for both). SBP was elevated in both preeclamptic groups (Group 3 and 4) compared with normotensive groups (Group 1 and 2), with no statistically significant difference between preeclamptic women with or without malaria. Diastolic blood pressure followed a similar trend, with a progressive increase observed from normotensive women without malaria to preeclamptic women without malaria (Table 1).

**Table 1:** Sociodemographic characteristics of study participants.

**Variables**	**Group 1 (** **n** = 50**)**	**Group 2 (** **n** = 50**)**	**Group 3 (** **n** = 50**)**	**Group 4 (** **n** = 50**)**	**p** **value**
Maternal age(years), mean ± SD	28.97 ± 5.94	30.31 ± 5.99	31.20 ± 5.25	30.23 ± 6.79	0.184
Gestational age (weeks), mean ± SD	22.96 ± 5.29^a^	25.49 ± 5.87^ab^	25.87 ± 7.40^b^	24.77 ± 6.16^ab^	0.030
Gravidity (median, IQR)	2 (–3)	3 (2–3)	3 (2–4)	3 (2–3)	0.070
Parity (median, IQR)	2 (1– 3)	2 (1–3)	2 (1– 3)	2 (1–3)	0.193
Primigravida, n (%)	13 (26.0%)	7 (14.0%)	9 (18.0%)	12 (24.0%)	0.082
BMI (Kg/m^2^), mean ± SD	28.10 ± 4.90	28.03 ± 3.98	27.02 ± 5.21	26.76 ± 3.87	0.184
SBP (mmHg), mean ± SD	110.60 ± 7.50^a^	109.11 ± 9.49^a^	152.83 ± 9.65^b^	151.16 ± 8.52^b^	<0.001
DBP (mmHg), mean ± SD	65.39 ± 7.94^a^	74.57 ± 11.34^b^	98.06 ± 6.75^c^	100.14 ± 5.31^c^	<0.001

*Note:* Group 1 (controls), normotensive without malaria; Group 2, normotensive with malaria; Group 3, preeclamptic with malaria; Group 4, preeclamptic without malaria. Superscripts (a, b, c) denote groups that are significantly different from one another (*p* < 0.05) (Tukey HSD post hoc comparisons). Groups sharing the same superscript are not significantly different.

Abbreviations: BMI, body mass index; DBP, diastolic blood pressure; IQR, interquartile range; SD, standard deviation; SBP, systolic blood pressure.

### 3.2. Inflammatory and Complement Biomarker Profiles

Plasma concentrations of inflammatory and complement‐related biomarkers were statistically different across the four groups (*p* < 0.001). Elevated levels of adipsin, C3a, C5a, TNF‐*α* and IL‐8 were found in both preeclamptic groups (with and without malaria) and the normotensive group with malaria compared with the controls. The highest concentrations of adipsin, C5a, TNF‐*α* and IL‐6 were observed in women with preeclampsia coexisting with malaria (Table 2).

**Table 2:** Plasma concentrations of adipsin, inflammatory and complement biomarkers amongst study participants.

**Variables**	**Group 1 (** **n** = 50**)**	**Group 2 (** **n** = 50**)**	**Group 3 (** **n** = 50**)**	**Group 4 (** **n** = 50**)**	**p** **value**
Adipsin (pg/mL), (median, IQR)	169.10 (138.20–337.70)	2918.00 (2375.00–3568.50)	4149.50(3520.20–4361.08)	2305.00(1507.50–3607.50)	< 0.001
C3a (ng/ml), (median, IQR)	207.00 (165.50–273.75)	274.00 (204.00–358.50)	435.00 (368.50–521.25)	286.60 (252.75–344.25)	< 0.001
C5a (ng/ml), (median, IQR)	8.38 (6.49–11.09)	31.60 (25.13–44.80)	62.60 (53.37–71.80)	23.10 (16.98–31.55)	< 0.001
TNF‐*α* (pg/mL), (median, IQR)	0.08 (0.06–0.10)	0.15 (0.10–0.24)	0.62 (0.54–0.74)	0.22 (0.17–0.26)	< 0.001
IL‐6 (pg/mL), (median, IQR)	0.49 (0.24–0.74)	0.35 (0.25–0.43)	0.65 (0.60–0.73)	0.30 (0.18–0.49)	< 0.001
IL‐8 (pg/mL), (median, IQR)	0.25 (0.18–0.33)	1.32 (0.71–2.42)	0.36 (0.18–0.82)	0.56 (0.27–0.75)	< 0.001
IFN‐*γ* (pg/mL), (median, IQR)	0.16 (0.13–0.20)	2.39 (1.61–3.20)	0.81 (0.39–1.35)	0.10 (0.07–0.13)	< 0.001

*Note:* Group 1 (controls), normotensive without malaria; Group 2, normotensive with malaria; Group 3, preeclamptic with malaria; Group 4, preeclamptic without malaria.

Abbreviations: C3a, complement component 3a; C5a, complement component 5a; IFN‐*γ*, interferon‐gamma; IL‐6, interleukin‐6; IL‐8 = interleukin‐8; IQR, interquartile range; TNF‐*α*, tumour necrosis factor‐alpha.

Pairwise comparisons amongst the groups showed no statistically significant differences between normotensive with malaria and preeclamptic without malaria for adipsin (*p* = 0.170), C3a (*p* = 0.357) and IL‐6 (*p* = 0.419) (Figure 1).

Figure 1Plasma concentrations of (a) adipsin, (b) C3a and (c) IL‐6 across groups: Group 1 (normotensive without malaria), Group 2 (normotensive with malaria), Group 3 (preeclamptic with malaria) and Group 4 (preeclamptic without malaria). Each boxplot depicts the median, interquartile range (IQR) and outliers.

**Figure:**
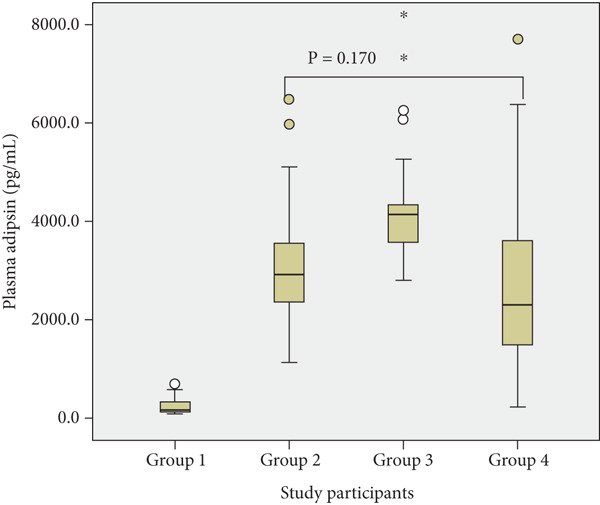
(a) Adipsin Levels

**Figure:**
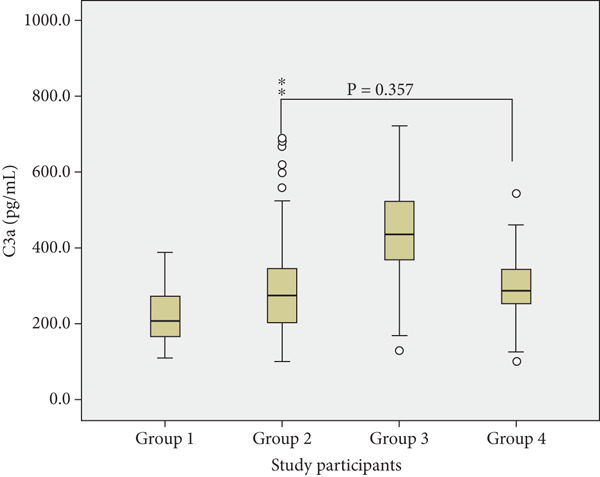
(b) C3a Levels

**Figure:**
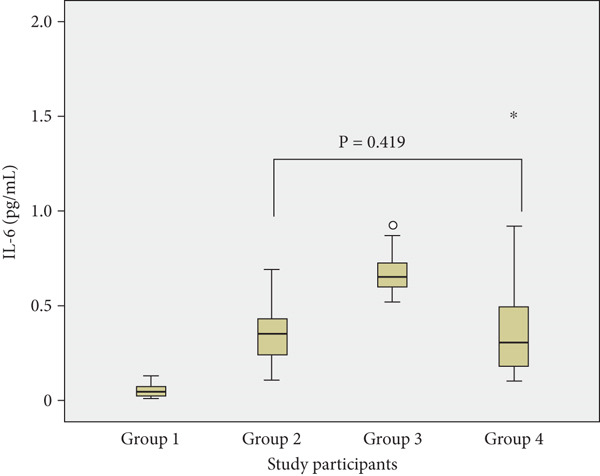
(c) IL‐6 Levels

### 3.3. Independent and Interactive Effects of Preeclampsia and Malaria on Plasma Adipsin Concentrations

Multivariable linear regression was performed to evaluate the independent and interactive effects of preeclampsia and malaria on plasma adipsin levels. After adjusting for maternal age, gestational age and BMI, the overall model was statistically significant (F (6193) = 33.75, *p* < 0.001), accounting for 42.6% of the variance in adipsin concentrations (*R* ^2^ = 0.426, adjusted *R* ^2^ = 0.413).

**Figure 2:**
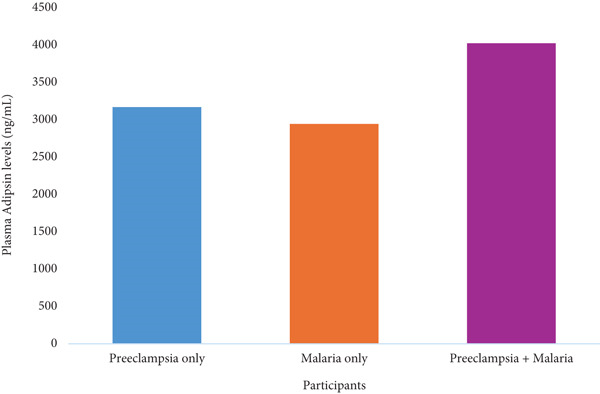
Interaction effect of preeclampsia and malaria on plasma adipsin levels.

Both preeclampsia and malaria status were independently associated with significantly elevated plasma adipsin levels. Preeclampsia increased adipsin concentrations by an average of 3168.76 ± 305.70 pg/mL, *p* < 0.001, whereas malaria increased adipsin levels by 2943.05 ± 304.37 pg/mL, *p* < 0.001. A significant negative interaction was observed between preeclampsia and malaria (*B* = –2087.83, ± 425.89, *p* < 0.001) (Table 3, Figure 2).

**Table 3:** Regression analysis of the independent and interactive effects of preeclampsia and malaria on plasma adipsin Concentrations.

**Predictor**	**Unstandardised coefficient**		**Standardised coefficients**	**p** **value**
	**B**	**Std error**	**Beta**	
Preeclampsia	3168.76	305.70	0.684	< 0.001
Malaria	2943.05	304.37	0.635	< 0.001
Interaction (PE × malaria)	−2087.83	425.89	−0.390	< 0.001

### 3.4. Correlation Between Plasma Adipsin and Malaria Parasite Density, Inflammatory and Complement Biomarkers

**Figure 3:**
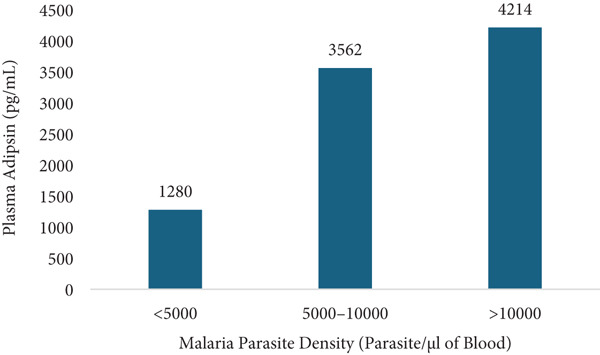
Plasma adipsin concentrations by malaria parasite density.

Plasma adipsin levels showed significant positive correlations with parasite density and all measured biomarkers. The strongest correlations were observed with C5a (*ρ* = 0.695, *p* < 0.001), IL‐6 (*ρ* = 0.687, *p* < 0.001), and TNF‐*α* (*ρ* = 0.645, *p* < 0.001). Moderate correlations were also observed with malaria parasite density (*ρ* = 0.553, *p* < 0.001), IL‐8 (*ρ* = 0.475, *p* < 0.001) and C3a (*ρ* = 0.437, *p* < 0.001) (Table 4). Furthermore, adipsin levels increased progressively with malaria parasite density. The median levels of adipsin were lowest in the low‐parasitaemia group and highest in women with high parasitaemia (Figure 3).

**Table 4:** Spearman′s rank correlation between plasma adipsin and malaria parasite density, complement components and pro‐inflammatory cytokines.

**Variables**	**Spearman′s *ρ***	**p** **value**
Malaria parasite density (parasites/*μ*l blood)	0.553	< 0.001
C3a (ng/ml)	0.437	< 0.001
C5a (ng/ml)	0.695	< 0.001
TNF‐*α* (pg/mL)	0.645	< 0.001
IL‐6 (pg/mL)	0.687	< 0.001
IL‐8 (pg/mL)	0.475	< 0.001
IFN‐*γ* (pg/mL)	0.365	0.022

Abbreviations: C3a, complement component 3a; C5a, complement component 5a; IFN‐*γ*, interferon‐gamma; IL‐6, interleukin‐6; IL‐8, interleukin‐8; TNF‐*α*, tumour necrosis factor‐alpha.

In a binary logistic regression model, higher malaria parasite density was independently associated with elevated plasma adipsin levels (OR = 2.32, 95% CI: 1.30–4.13, *p* = 0.003). Amongst the inflammatory biomarkers, TNF‐*α* was the only cytokine significantly associated with high adipsin concentrations (OR = 1.063, 95% CI: 1.027–1.099, *p* < 0.001). Complement components C3a (OR = 1.002, 95% CI: 1.000–1.005, *p* = 0.010) and C5a (OR = 1.018, 95% CI: 1.010–1.028, *p* < 0.001) also demonstrated significant independent associations with elevated plasma adipsin after adjustment for all covariates (Table 5).

**Table 5:** Adjusted odds ratios for predictors of elevated plasma adipsin.

**Variables**	**OR (95% CI)**	**p** **value**
Malaria parasite density (parasites/*μ*l blood)	2.32 (1.30–4.13)	0.003
C3a (ng/ml)	1.002 (1.000–1.005)	0.010
C5a (ng/ml)	1.018 (1.010–1.028)	< 0.001
TNF‐*α* (pg/mL)	1.063 (1.027–1.099)	< 0.001
IL‐6 (pg/mL)	3.05 (0.60–5.58)	0.181
IL‐8 (pg/mL)	0.94 (0.61–1.45)	0.772
IFN‐*γ* (pg/mL)	0.99 (0.68–1.44)	0.994

Abbreviations: C3a, complement component 3a; C5a, complement component 5a; IFN‐*γ*, interferon‐gamma; IL‐6, interleukin‐6; IL‐8 = Interleukin‐8; TNF‐*α*, tumour necrosis factor‐alpha.

### 3.5. Diagnostic Accuracy of Plasma Adipsin for Detecting Preeclampsia

**Figure 4:**
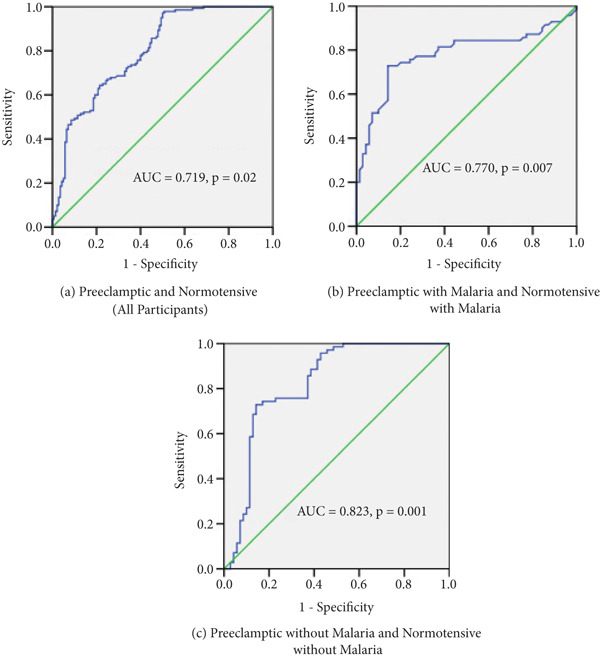
Receiver operating characteristic (ROC) curves showing the diagnostic performance of plasma adipsin across subgroups.

ROC curve analysis showed variability in the diagnostic performance of plasma adipsin across different groups. In the overall cohort of participants with preeclampsia and those who are normotensive, the AUC was 0.719 (*p* = 0.02). A cut‐off level of 756.7 pg/mL resulted in a sensitivity of 97.1% and a specificity of 50.0%. For participants with malaria, plasma adipsin demonstrated an AUC of 0.770 (*p* = 0.007), with a sensitivity of 85.7% and a specificity of 62.1% at a threshold of 1834.5 pg/mL. The highest diagnostic accuracy was observed in the subgroup without malaria (AUC = 0.823, *p* < 0.001). In this group, a cut‐off level of 509.6 pg/mL provided a sensitivity of 82.9% and a specificity of 87.9% (Table 6, Figure 4).

**Table 6:** Diagnostic performance of plasma adipsin for detecting preeclampsia across subgroups.

**Participants**	**Cutoff (pg/mL)**	**Sensitivity (%)(95% CI)**	**Specificity (%)(95% CI)**	**AUC(95% CI)**
Preeclamptic and normotensive (all participants)	756.70	97.10 (93.87–99.56)	50.00 (44.74–57.87)	0.719 (0.688–0.781)
Preeclamptic with malaria and Normotensive with malaria	1834.50	85.70 (77.28–92.31)	62.10 (58.42–72.31)	0.770 (0.748–0.850)
Preeclamptic without malaria and Normotensive without malaria	509.60	82.90 (75.02–86.91)	87.90 (83.79–94.42)	0.823 (0.803–0.902)

## 4. Discussion

This study assessed the relationship between malaria infection, adipsin levels, and preeclampsia. The maternal age, gravidity, parity, BMI and primigravida status did not show any statistically significant difference across the four groups. This is important for interpreting the immunological and biomarker data, as it reduces the potential confounding effects of reproductive history, BMI, or age‐related immunological changes.

Although systolic and diastolic blood pressures were significantly elevated in the preeclamptic groups compared with normotensive controls, no significant difference was observed between preeclamptic women with and without malaria infection. This indicates that the observed differences in plasma adipsin levels are less likely to be attributed to variations in the severity of hypertension. This aligns with the best practises in biomarker research, ensuring that the results are more reliable and generalizable [18, 19].

Plasma levels of adipsin, C3a, C5a, TNF‐*α* and IL‐8 differed significantly across the study groups. Both preeclampsia and malaria were independently associated with elevated levels of these biomarkers when compared with normotensive controls. These findings support the role of complement activation and systemic inflammation in the pathogenesis of both conditions [20, 21].

The elevation of plasma adipsin in both preeclamptic and malaria‐infected women is consistent with its biological role as a key enzyme in the alternative complement pathway [22]. The markedly elevated levels of adipsin in women with both conditions may reflect a state of excessive or unregulated complement activation [23, 24]. This finding corroborates earlier studies that have reported elevated complement activation in both preeclampsia and *P. falciparum* infection [8, 15, 25]. However, the circulating adipsin concentrations in our study population are higher than those reported in predominantly European or North American cohorts [26, 27]. This variation likely reflects population‐specific metabolic and immunological characteristics, including a higher burden of metabolic syndrome, chronic low‐grade inflammation [28, 29] and regional exposure to infections such as malaria, which are known to augment complement pathway activity during pregnancy [25].

There was no statistically significant difference in the levels of plasma adipsin between the normotensive malaria‐infected group and the preeclamptic group without malaria. This suggests that malaria may induce a complement and inflammatory response of similar magnitude to that observed in preeclampsia. This finding has important implications for the diagnostic specificity of plasma adipsin as a biomarker for preeclampsia in malaria‐endemic settings. It may be advisable to screen pregnant women for malaria infection when plasma adipsin is being considered for use as a diagnostic marker in these regions.

Plasma adipsin levels showed strong positive correlations with C5a, IL‐6 and TNF‐*α*. These associations are also consistent with adipsin′s role in the alternative complement pathway, which is activated in both malaria and preeclampsia as reported by Mcdonald et al. [25] and Regal et al. [8]. The strength of these correlations suggests that adipsin secretion is at least partly regulated by inflammatory and complement‐mediated immune activity, rather than solely by placental or hypertensive stress [12, 30]. Similar findings have been reported by studies conducted in the Kenyan and Cameroonian populations [2, 31].

Moderate but significant correlations were also observed between plasma adipsin levels and malaria parasite density, IL‐8 and C3a. This suggests that increased parasitaemia, associated with systemic complement activation and cytokine release [32], may drive adipsin expression. This is further supported by the increase in median adipsin levels with rising malaria parasite density (Figure 3), suggesting a parasite‐density–dependent upregulation of adipsin secretion.

This study further demonstrates that both preeclampsia and malaria independently elevate plasma adipsin levels, with a significant negative interaction observed when both conditions coexist. This interaction suggests a nonadditive effect, likely due to shared inflammatory or complement pathways [1, 33]. The significant negative interaction between preeclampsia and malaria on adipsin levels suggests that the combined effect of these conditions on adipsin concentration is less than the sum of their individual effects. This may alter adipsin′s diagnostic threshold in individuals who present with both conditions, limiting its standalone diagnostic value in malaria‐endemic settings. These findings highlight the need for stratified thresholds and suggest that adipsin may be more effective when used alongside other biomarkers or in malaria‐negative populations.

The findings from this study also demonstrate that the diagnostic utility of plasma adipsin as a biomarker for preeclampsia is influenced by the presence of malaria infection. In the entire cohort, adipsin showed moderate discriminative ability (AUC = 0.719), with a high sensitivity (97.1%) but low specificity (50.0%) at a cut‐off of 756.7 pg/mL. This likely reflects false positives due to elevated adipsin levels in malaria‐infected participants. This pattern contrasts with the findings of Liu et al. [11] who reported an AUC of 0.83 with a sensitivity and specificity of 81.8% and 75.8%, respectively, in nonendemic settings.

Subgroup analysis revealed that plasma adipsin achieved its highest diagnostic accuracy in women without malaria (AUC = 0.823), with a sensitivity of 82.9% and specificity of 87.9% at a lower threshold of 509.6 pg/mL. In the malaria‐infected subgroup, however, adipsin′s diagnostic performance was reduced (AUC = 0.770), and the diagnostic threshold increased significantly to 1834.5 pg/mL. This suggests that in the absence of malaria‐related immune activation, plasma adipsin performs better as a biomarker for preeclampsia. The elevated diagnostic threshold required to distinguish preeclamptic women with malaria from their normotensive counterparts (cutoff = 1834.5 pg/mL) compared with that required in the nonmalaria subgroup (cutoff = 509.6 pg/mL) reflects the significant impact of malaria on baseline adipsin levels. These findings align with those of Santiago et al. [15] and support the hypothesis that malaria‐induced complement activation influences circulating plasma adipsin levels. These results highlight the importance of considering coexisting conditions, such as malaria, when using adipsin as a screening or diagnostic biomarker for preeclampsia, especially in populations where malaria is prevalent.

The results from this study suggest that whilst plasma adipsin has diagnostic potential, its clinical utility as a biomarker for preeclampsia in malaria‐endemic settings must be approached with caution. Diagnostic thresholds may need to be context‐specific, and complementary biomarkers may be required to improve specificity. Future studies may explore multiple biomarker algorithms or malaria‐adjusted cut‐offs to optimise diagnostic performance in malaria‐endemic environments.

Despite these promising results, the study has some limitations. It was conducted in a single geographic region, which may limit the generalisability of the results to other malaria‐endemic populations. Although malaria diagnosis was confirmed by microscopy, submicroscopic parasitaemia and anaemia, both of which are known to influence immune and complement activation were not assessed.

## 5. Conclusion

This study demonstrates that malaria infection significantly influences plasma adipsin levels in preeclampsia through the activation of complement and inflammatory pathways. These findings highlight the need for malaria‐adjusted diagnostic thresholds and context‐specific validation of adipsin as a biomarker in malaria‐endemic regions.

Policymakers should consider integrating routine malaria screening into preeclampsia diagnostic pathways and prioritise local validation studies before incorporating adipsin‐based diagnostics into antenatal screening programmes. Such measures will improve the accuracy of preeclampsia detection and contribute to better maternal health outcomes in this population.

**Nomenclature**
ISSHP: International Society for the Study of Hypertension in Pregnancy
ELISA: enzyme‐linked immunosorbent assay
HIV: human immunodeficiency virus
K₂‐EDTA: dipotassium ethylenediaminetetraacetic acid
C3a: complement component 3a
C5a: complement component 5a.
TNF‐*α*
: tumour necrosis factor‐alpha
IL‐6: interleukin‐6
IL‐8: interleukin‐8
IFN‐*γ*
: interferon‐gamma
HRP2: histidine‐rich protein 2
pLDH: plasmodium lactate dehydrogenase
WBC: white blood cell
BMI: body mass index
SBP: systolic blood pressure
DBP: diastolic blood pressure

## Disclosure

All authors read and approved the final manuscript.

## Conflicts of Interest

The authors declare no conflicts of interest.

## Author Contributions

B.O.M. and E.O.A. conceptualised the research idea; L.A.F. and B.O.M. wrote the experiment protocols; L.A.F. provided technical guidance on laboratory assays; E.O.A. and A.O.M. conducted the experiments and data collection; B.O.M. and E.O.A. contributed to data analysis; B.O.M. wrote the manuscript; L.A.F. and A.O.M. reviewed the manuscript.

## Funding

No funding was received for this manuscript.

## Data Availability

The data that support the findings of this study are available from the corresponding author upon reasonable request.
